# Expression Data Analysis to Identify Biomarkers Associated with Asthma in Children

**DOI:** 10.1155/2014/165175

**Published:** 2014-03-27

**Authors:** Wen Xu

**Affiliations:** Department of Paediatrics, Rizhao City People's Hospital, No. 126 Donggang Area, Tai'an Road, Rizhao City, Shandong 276800, China

## Abstract

Asthma is characterized by recurrent episodes of wheezing, shortness of breath, chest tightness, and coughing. It is usually caused by a combination of complex and incompletely understood environmental and genetic interactions. We obtained gene expression data with high-throughput screening and identified biomarkers of children's asthma using bioinformatics tools. Next, we explained the pathogenesis of children's asthma from the perspective of gene regulatory networks: DAVID was applied to perform Kyoto Encyclopedia of Genes and Genomes (KEGG) pathway enriching analysis for the top 3000 pairs of relationships in differentially regulatory network. Finally, we found that HAND1, PTK1, NFKB1, ZIC3, STAT6, E2F1, PELP1, USF2, and CBFB may play important roles in children's asthma initiation. On account of regulatory impact factor (RIF) score, HAND1, PTK7, and ZIC3 were the potential asthma-related factors. Our study provided some foundations of a strategy for biomarker discovery despite a poor understanding of the mechanisms underlying children's asthma.

## 1. Introduction

Asthma is the most common chronic inflammatory disease of the trachea in childhood characterized by variable and recurring symptoms, reversible airflow obstruction, and bronchospasm [1]. There are significant variations in prevalence in different regions and ethnics; generally, a developed country has a higher prevalence than a developing country. Asthma prevalence is rising worldwide now, and according to the report of International Children's Asthma and Allergic Organization (ISAAC), the incidence rate of British children's asthma rose from 10.2% at 2000 to 20.9% at 2011 [2]; the prevalence in American children below 17 years increased from 3.2% at 1999 to 5.7% at 2010 [3]. In China, the incidence rate of urban children aged 0–14 increased from 0.5% at 1998 to 4.33% at 2008 [4]. Thus, there is an urgent need to identify the underlying basis of asthma.

Asthma is thought to be caused by a combination of genetic and environmental factors [5], which influence both the severity and responsiveness of asthma in treatment [6]. Smoking during pregnancy and after delivery [7], low air quality, and exposure to indoor allergens [8], such as dust mites, cockroaches, animal dander, and mold, have been found to be associated with children's asthma. Asthma is believed to have a strong genetic background, and hundreds of genes have been identified to be related with asthma, including GSTM1, IL10, CTLA-4, SPINK5, LTC4S, IL4R, and ADAM33 [9]. Some genetic variants may cause asthma only when they are combined with specific environmental exposures [10], for example, a specific single nucleotide polymorphism in the CD14 region and exposure to endotoxin [11]. Understanding the genetic basis of asthma susceptibility will allow disease prediction and risk stratification [12].

Bioinformatics plays an important role in addressing the complexity of the underlying genetic basis of common human disease [13]. Microarray data analysis enables the identification of disease marker genes and gene regulatory networks [14, 15]. In this study, we obtained the gene expression profiles using high-throughput technology and screened differentially coexpressed gene pairs. The availability and integration of high-throughput gene expression data with computational bioinformatics analysis may shed new lights into molecular biomarker identification of children's asthma.

## 2. Materials and Methods

### 2.1. Data Source and Preprocessing

The expression profile of GSE18965 [16] was downloaded from Gene Expression Omnibus-GEO database (http://www.ncbi.nlm.nih.gov/geo/) of NCBI (National Center of Biotechnology Information) based on GPL96 [HG-U133_Plus_2] Affymetrix Human Genome U133 Plus 2.0 Array. Seven normal tissues' microarray and nine children's asthma tissues' microarray were available. Then, probes in expression profile were transformed to corresponding symbols based on GPL96 platform. For genes related to many probes, the average expression value was calculated as the only symbol, and there were 13,046 gene symbols after transformation. Next, limma package in R language was used to screen the differentially expressed genes (DEGs), and false discovery rate (FDR) < 0.05 was set as the threshold.

### 2.2. Screening of Transcriptional Regulatory Relationships

According to the central dogma, approaches resulting in gene expression differences are varied, but on transcription level, regulatory molecules are the decisive factors, for example, transcription factors (TFs), which regulate the turn on and off of genes. Firstly, human h18 transcription factor binding sites data and genetic coordinate position information were downloaded from the UCSC database [17]. Secondly, we searched transcription factor binding sites between the range of 1 kb upstream and 0.5 kb downstream in the transcription start site of each gene, and the found TF was considered to be associated with this gene, and finally we got 214,608 pairs of gene regulatory relationships on 216 TFs for 16,863 genes.

### 2.3. Differentially Coexpressed Analysis [18]

Differentially coexpressed analysis determines the discrepancy in coexpression of gene pairs or gene-TF pairs under different conditions [19]. Previous differentially coexpressed analyses have revealed many insightful biological hypotheses. In this study, for any pair of genes or pair of gene versus TF (*X*, *Y*), the Pearson correlation coefficient (PCC) in normal tissues (P-normal) and tumor tissues (P-tumor) was calculated, and then their absolute difference was obtained. Finally, the pairs with absolute difference >1 were selected as differentially coexpressed pairs. There were two kinds of coexpression relationships: negative, when P-normal ∈[−1,0]; positive, when P-tumor ∈[−1,0] and vice versa. Consider

(1) $$\begin{array}{c} \text{Diff}=\text{abs}\;\left(r1_{ij}-r2_{ij}\right). \end{array}$$

In this term, *r*1_*ij*_ represents PCC of gene/TF *i* and gene/TF *j* at normal state; *r*2_*ij*_ represents the PCC of gene/TF *i* and *j* at tumor state.

### 2.4. Regulatory Impact Factors (RIF) Calculation

Regulatory impact factor (RIF) appears to be a robust and valuable methodology to identify the regulators with the highest contribution to differential gene expression in two biological conditions. It is a metric given to each TF that combines the expression values of target genes and the coexpression values of TFs and the target genes. The measures of RIF are computed as follows [20]:

(2) $$\begin{array}{c} \text{RI}{\text{F}}_{i}=\frac{1}{n_{\text{de}}}\sum_{j=1}^{j=n_{\text{de}}}\left[{\left(e1_{j}\times r1_{ij}\right)}^{2}-{\left(e2_{j}\times r2_{ij}\right)}^{2}\right], \end{array}$$

where *n*_de_ is the number of DEGs; *e*1_*j*_ and *e*2_*j*_ represent the expression values of the DEG *j* in conditions 1 and 2, respectively; *r*1_*ij*_ and *r*2_*ij*_ represent the coexpression correlation between the TF *i* and the DEG *j* in conditions 1 and 2, respectively.

### 2.5. Pathway Enrichment Analysis

In order to facilitate the functional annotation and analysis of large lists of genes in the regulatory network, we inputted all the DEGs into DAVID for KEGG term enrichment analysis. The DAVID enriches canonical pathways by calculating the association between a given set of genes and a canonical pathway using hypergeometric test [21]. A *P* value <0.05 was the screening criterion.

## 3. Results

### 3.1. Screening of Differentially Coexpressed Gene Pairs

If the expressions of two genes or gene versus TF in a series of samples are similar, they are called coexpression pairs. If the pairs are coexpressed in condition A, but not in condition B, or vice versa, then they are called the differentially coexpressed genes pairs. We calculated the PCCs between two genes and gene versus TF with their expression profile data at normal and tumor stages and then used formula (1) to screen differentially coexpressed gene pairs. A total of 9,775,369 differentially coexpressed genes pairs were obtained (Table 1).

### 3.2. Construction and Analysis of Differentially Regulatory Network

**Figure 1:**
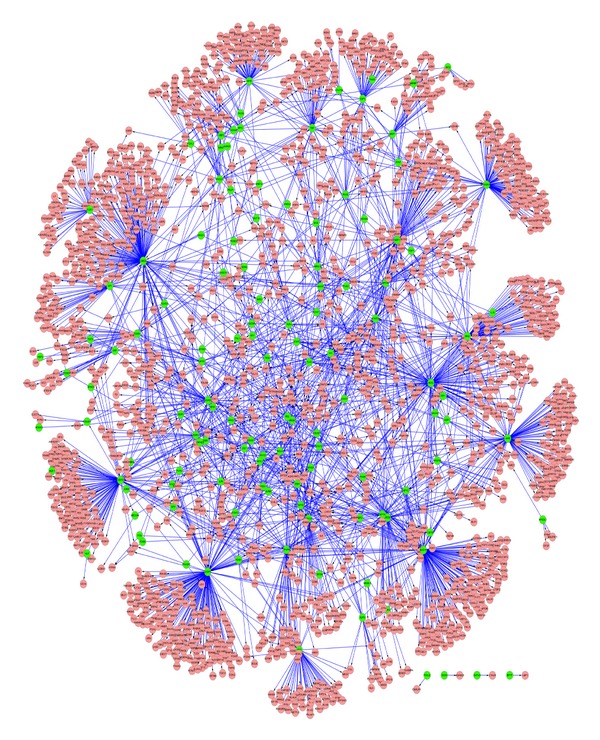
Differentially regulatory network (showing the top 25% relationships). Green nodes represent TFs, pink nodes represent target genes, and lines represent the regulation relationships between them.

Transcriptional regulation pairs were selected based on the selected differentially coexpressed gene pairs and then were used for the construction of differentially regulatory network. The transcriptional regulation relationship in the network under disease states was different from that under normal state, which may possess a significant impact on the incidence of disease. The constructed differentially regulatory network was comprised of 10,899 pairs of regulation relationships, including 133 TFs and 5,083 target genes. The top 25% relationships were visualized using Cytoscape software (Figure 1).

### 3.3. Impact Analysis of Transcription Factor

The above network generated vast amounts of data. In order to focus on the most meaningful information, we evaluated the impact of TFs by calculating their RIF. The top 10 ranked TFs with higher RIF were HAND1, PTK1, NFKB1, ZIC3, STAT6, E2F1, PELP1, USF2, CBFB, SOX9, and FOXO4 (Table 2).

By searching PubMed, NFKB1 [22, 23], STAT6 [24], E2F1 [25], USF1 [26], and CBFB [21] were the verified asthma-related TFs, while NFKB1 [22, 23] and STAT6 [24] were the newly discovered asthma-related genes in 2013. In addition, HAND1, PTK7, and ZIC3 were found to be potential asthma-related factors considering their TIF values.

### 3.4. Enrichment of KEGG Pathway

We used DAVID to perform KEGG pathway enriching analysis for the top 3000 pairs of relationships in differentially regulatory network. As shown in Table 3, the differentially regulatory network was mainly enriched in some important pathways, such as cancer pathway, Wnt, and MAPK pathway.

## 4. Discussion

Molecular biomarkers are useful in improving diagnostic and prediction accuracy in clinic and treatment efficacy. Since microarray can interrogate expression levels of thousands of genes in human genome simultaneously, it has been widely used in the discovery of disease biomarkers [27–29]. In this work, we analyzed gene expression data with computational methods with the aim of uncovering biomarkers that were potentially dysregulated in children's asthma. We identified a total of 9,775,369 differentially coexpressed gene pairs between normal tissue microarray and children's asthma tissue microarray. After regulatory network construction and RIF analysis, we found that the TFs: HAND1, PTK1, NFKB1, ZIC3, STAT6, E2F1, PELP1, USF2, CBFB, SOX9, and FOXO4 may play important roles in children's asthma initiation. On account of RIF score, HAND1, PTK7, and ZIC3 were considered as potential asthma-related factors.

Heart and neural crest derivatives-expressed protein 1 (HAND 1) is a protein encoded by the HAND1 gene in human [30]. The protein encoded by this gene belongs to the basic helix-loop-helix family of TFs. A recent study provides evidence that HAND1 is indeed an important regulator of the interventricular boundary [31], but the role of HAND 1 in asthma has not been reported. Tyrosine-protein kinase-like 7 (PTK7) is a human enzyme encoded by the PTK7 gene [32]. Receptor protein tyrosine kinases could transduce extracellular signals across the cell membrane, and PTK7 is thought to mediate signals by recruiting other signaling molecules as defective receptor tyrosine kinases [33]. Our research showed that PTK7 gene was association with the occurrence of asthma in children. Zinc finger protein ZIC 3 is a protein encoded by the ZIC3 gene [34], which encodes a member of the ZIC family of C2H2-type zinc finger proteins. Our results highlight a role of Zic3 in the maintenance of asthma. However, further experimental verification is needed on the possible roles of HAND1, PTK7, and ZIC3 in asthma proposed in this study.

NFKB1, STAT6, and E2F1 were the verified asthma-related TFs in PubMed, and they were discovered to exert regulatory impact in this study. NFKB1 (nuclear factor of kappa light polypeptide gene enhancer in B cells 1), which located within the linkage peak, encodes the p105/p50 subunit of the NF*κ*B family of proteins [35]. By detecting the RNA expression in buccal mucosa samples of patients with asthma, NFKB1 was found to be differentially expressed [23]. NFKBIA/I*κ*B*α* is identified to be a central hub in transcriptional responses of prevalent childhood lung diseases, including asthma [22]. STAT6 gene (human signal transducer and activator of transcription 6) is considered as one of the most promising candidate genes for asthma [36]. Genomewide association studies have revealed that special polymorphism haplotype variants and epigenetic modifications of STAT6 are associated with asthma in childhood [24]. The transcription factor E2F1 is an additional target of c-Myc promoting cell cycle progression [37]. E2F1 was differentially expressed in asthma-diagnosed human donor lung tissues compared with normal bronchial epithelial cells [38].

During cellular processes, genes interact with each other; thus, disease-related genes may form differential coexpression patterns with other genes in different conditions. Most of the previous analysis applied a single gene differential expression method, whereas we applied differential coexpression analysis. The differential coexpression approach provides a FDR controlled list of interesting gene sets, with no requirement that genes be highly correlated in at least one biological condition, and it is now readily applied to data from individual and multiple experiments. Nevertheless, the differential coexpression gene pairs identified using the computational bioinformatics method should be further confirmed by in vitro analysis with normal controls.

In conclusion, our analysis identified 9,775,369 differentially coexpressed genes pairs associated with asthma initiation using a computational bioinformatics analysis of gene expression. We also uncovered a network of transcription factors that putatively contribute to the dysregulation of several genes in asthma. In the differentially regulatory network, transcription factors HAND1, PTK1, NFKB1, ZIC3, STAT6, E2F1, PELP1, USF2, CBFB, SOX9, and FOXO4 were found to have altered expression levels in asthma patients. We suggested that HAND1, PTK7, and ZIC3 may be used as biomarkers for asthma; however, more work is needed to validate our result.

## Figures and Tables

**Table 1:** Some differentially coexpressed gene pairs.

Gene1	Gene2	Diff.
ATF6	AACS	1.198925
MYCN	AAGAB	1.031
DDIT3	AAMP	1.237609
RREB1	AAMP	1.005331
STAT2	AAMP	1.085082
STAT3	AAMP	1.268363
STAT4	AAMP	1.067587
CBFB	AASS	1.027421
NFYA	AASS	1.028667
ARNT	AATF	1.000478

**Table 2:** Top 10 TFs in differentially regulatory network.

TF	RIF_score	RIF_rank
HAND1	3.675835	1
PTK7	3.646741	2
NFKB1	3.341134	3
ZIC3	3.321142	4
STAT6	3.301687	5
E2F1	3.206273	6
PELP1	3.051003	7
USF2	3.037221	8
CBFB	2.996446	9
SOX9	2.968472	10
FOXO4	2.837118	11

RIF: regulatory impact factors.

**Table 3:** Enriched KEGG pathways of differentially regulatory network.

Category	Term	FDR (%)
KEGG_PATHWAY	hsa05200: pathway in cancer	3.26*E* − 07
KEGG_PATHWAY	hsa04010: MAPK signaling pathway	0.001322
KEGG_PATHWAY	hsa05221: acute myeloid leukemia	0.040656
KEGG_PATHWAY	hsa04520: adherens junction	0.062934
KEGG_PATHWAY	hsa04310: Wnt signaling pathway	0.092027
KEGG_PATHWAY	hsa05215: prostate cancer	0.120383
KEGG_PATHWAY	hsa05220: chronic myeloid leukemia	0.326993
KEGG_PATHWAY	hsa05210: colorectal cancer	0.77687
KEGG_PATHWAY	hsa04060: cytokine-cytokine receptor interaction	1.783078
KEGG_PATHWAY	hsa04330: Notch signaling pathway	2.388182
KEGG_PATHWAY	hsa04062: chemokine signaling pathway	2.592513
KEGG_PATHWAY	hsa04630: Jak-STAT signaling pathway	2.884224
KEGG_PATHWAY	hsa04350: TGF-beta signaling pathway	3.093238
KEGG_PATHWAY	hsa04720: long-term potentiation	3.63158
KEGG_PATHWAY	hsa04960: aldosterone-regulated sodium reabsorption	4.945282

FDR: false discovery rate.
